# Atypical behavior of NFATc1 in cultured intercostal myofibers

**DOI:** 10.1186/2044-5040-4-1

**Published:** 2014-01-03

**Authors:** Patrick Robison, Erick O Hernández-Ochoa, Martin F Schneider

**Affiliations:** 1 Department of Biochemistry and Molecular Biology, University of Maryland School of Medicine, 108 N Greene Street, Baltimore, MD 21201, USA

**Keywords:** NFATc1, Skeletal muscle, Respiratory muscle, Intercostal muscle, Excitation-transcription coupling

## Abstract

**Background:**

The NFATc transcription factor family is responsible for coupling cytoplasmic calcium signals to transcription programs in a wide variety of cell types. In skeletal muscle, these transcription factors control the fiber type in response to muscle activity. This excitation-transcription (E-T) coupling permits functional adaptation of muscle according to use. The activity dependence of these transcription programs is sensitive to the firing patterns of the muscle, not merely the period of activity, enabling a nuanced adaptation to various functional tasks.

**Methods:**

Isolated skeletal muscle fibers expressing exogenous fluorescent NFATc1 were studied by confocal microscopy under stimulation both with and without pharmacological inhibitors. Western blots of whole muscle lysates were also used.

**Results:**

This study investigates the activity dependent response of NFATc1 skeletal muscle fibers cultured from mice, comparing fibers of respiratory origin to muscles responsible for limb locomotion. Using patterns of stimulation known to strongly activate NFATc1 in the commonly cultured flexor digitorum brevis and soleus muscles, we have observed significant deactivation of NFATc1 in cultured intercostal muscle fibers. This effect is at least partially dependent on the action of JNK and CaMKII in intercostal fibers.

**Conclusions:**

Our findings highlight the role of lineage in the NFAT pathway, showing that the respiratory intercostal muscle fibers decode similar E-T coupling signals into NFAT transcriptional programs in a different manner from the more commonly studied locomotor muscles of the limbs.

## Background

The functional adaptability of skeletal muscle is an elegant regulatory phenomenon, coupling transcriptional adaptation to a particular function in the role to which the muscle is adapting. The cellular events coupling excitation and contraction with transcriptional activity are known collectively as excitation-transcription (E-T) coupling [1,2]. This process is perhaps best illustrated by cross-reinnervation [3,4] studies in which the target muscle begins to take on the properties of the muscle with which it has had its motor neurons exchanged. However, there is also a substantial effect of muscle lineage which limits the degree to which a muscle is capable of adapting to a new activity paradigm. This has been noted in adult muscle [5,6], but lineage effects can also be observed early in developmental stages [7].

**Figure 1:**
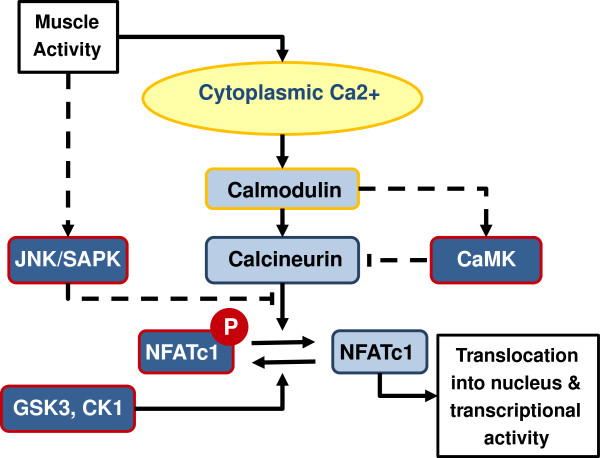
The activation and inactivation of NFATc1 in skeletal muscle fibers.

The NFATc transcription factors in skeletal muscle are primarily regulators of muscle type transformation [8-10]. NFATc1 splice variants are the dominant isoforms in skeletal muscle, expressed as several splice variants with very similar activation/inactivation domains [11]. Activation of NFATc1 is determined by the phosphorylation state of a serine rich region (SRR) including 13 regulatory phosphorylation sites [12] and at least three serine-proline repeats (SP repeats) forming a phosphorylation-dependent target for the isomerase Pin1 [13]. The phosphorylation of these sites controls a conformational change, exposing nuclear localization sequences when dephosphorylated and nuclear export sequences when phosphorylated [14]. This site is the subject of a complex dynamic equilibrium through which NFATc1 is regulated by kinases and phosphatases. At rest, the equilibrium is dominated by the kinases casein kinase 1 (CK1), glycogen synthase kinase 3 (GSK3), and dual-specificity tyrosine-phosphorylation regulated kinase (DYRK) [15], ensuring phosphorylation and therefore nuclear exclusion/transcriptional inactivity. During muscle activity, the calmodulin-dependent phosphatase calcineurin (CN) strips phosphates from the SRR/SP repeats and permits NFATc1 translocation and transcriptional activity (Figure 1). It should be noted that the activation of NFAT is not a broad response to calcium signaling. Indeed, it has been known for some time that the NFAT pathway is capable of distinguishing calcium signals of similar magnitude by frequency [9,10], an ability which is critical to appropriate adaptive responses.

Regulation of CN is primarily by relief of autoinhibition upon Ca^2+^ dependent binding to calmodulin (CaM) [16], although some additional Ca^+2^ dependence may be conferred by the B subunit [17]. Adjacent to the SRR of NFATc1 are two CN binding domains [11], responsible for targeting the Ca^2+^/CaM dependent phosphatase activity to the SRR, permitting the activation of the canonical NFATc1 activation pathway [10,14,15,18].

Muscle activity is also known to promote the activity of several protein kinases, providing additional layers of potential regulation. The c-Jun N-terminal kinases (JNK) and calmodulin dependent kinases (CaMK) may be particularly relevant. JNK, a MAPK, shows strong activation following activity in skeletal muscle [19,20]. Although JNK, like many MAPKs, is somewhat promiscuous, it is of relevance to the activation of NFATc1 by phosphorylation of sites in the CN binding domains flanking the SRR. This results in inhibition of the interaction of CN with the transcription factor and inactivation of NFATc1 [12].

CaMKII actually shares an activation mechanism with CN, and it is therefore unsurprising that it is activated by muscle activity [21]. This kinase has an established role in E-T coupling though the histone deacetylases [22] but also targets CN for phosphorylation at Ser197, thus inhibiting phosphatase activity [23].

In this study we report reversed NFATc1 signaling in isolated muscle fibers under identical stimulation depending on the muscle of origin. This behavior is at least partially dependent on the activity regulated kinases CaMKII and JNK. We propose that altered expression levels of these kinases and CN result in a change in the inhibition/activation balance of activity-dependent signaling in different muscles, accounting for this atypical behavior. These findings have substantial implications for the use of limb muscles as a general model for skeletal muscle plasticity and function and highlight the underlying diversity of function and adaptability in skeletal muscle.

## Methods

### Model system

Six- to eight-week-old female CD1 mice were euthanized and flexor digitorum brevis (FDB), soleus (Sol) and intercostal (ItC) muscles were removed. The procedures for isolating single muscle fibers have been previously described [24]. In brief, ItC and FDB muscles were dissected and enzymatically digested, then mechanically triturated with a polished glass pipette. Resulting fibers were maintained suspended in MEM supplemented with 10% FBS until plating on laminin coated dishes or use in releasable calcium experiments. Isolated fibers were kept in an incubator at 37°C under 5% CO_2_. Muscles for tissue lysates were lysed in Tissue Protein Extraction Reagent (Thermo Scientific, Wilmington, DE, USA with protease and phosphatase inhibitors and then stored at −80°C until ready for use.

### NFATc1-GFP

**Figure 2:**
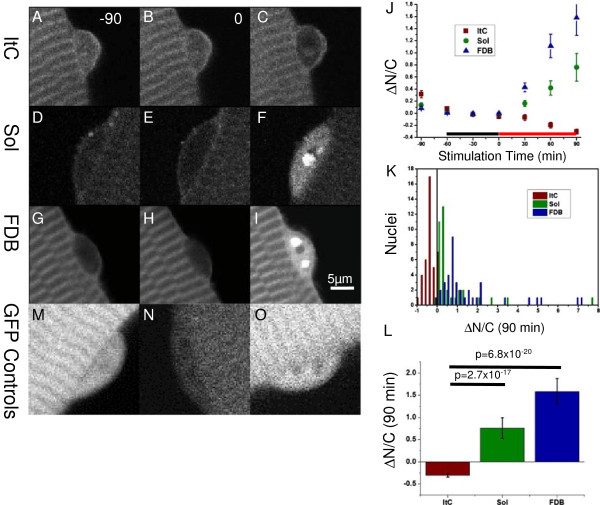
**Activity dependent inactivation of NFATc1.** Representative confocal images of intercostal (**A**-**C**; ItC), soleus (**D**-**F**; Sol) and flexor digitorum brevis (**G**-**I**; FDB) muscle fibers expressing NFATc1-GFP. The atypical response of intercostal fibers **(A-C)** compared to Soleus **(D-F)** and FDB **(G-I)** after stimulation is quantified by comparing baseline **(J**, black line) to the change in N/C ratio after 90 minutes of stimulation (**J**, red line). Although there is substantial heterogeneity in the responses of individual nuclei **(K)**, the overall behavior of nuclei in fibers from intercostal muscles is significantly different from either FDB or soleus **(L)**. Panels **M**, **N**, **O** show representative confocal images of ItC, Sol and FDB fibers expressing GFP. n = 40, 37, 38 nuclei from 15, 14, 25 fibers isolated from 4, 4, 3 animals for ItC, Sol and FDB respectively. Error bars show SEM.

The exogenous fusion protein NFATc1-GFP was expressed in isolated muscle fibers by exposure to an adenoviral construct [8] 24 hours following isolation as previously described [8,24]. After two or three days of expression (three to four days in culture), fibers were transferred to L-15 and imaged on an Olympus Fluoview 500 laser scanning confocal imaging system using an Olympus 60x/1.2 NA water immersion objective (Olympus, Center Valley, PA, USA). GFP images were recorded using an excitation wavelength of 488 nm and 505 nm long-pass emission filter. Following a minimum 30-minute period of acclimation, fibers were imaged at 30 minute intervals for one hour prior to start of stimulation and for one and a half hours after stimulation began (Figure 2J). Stimulation was carried out via platinum electrodes fitted to the dish so that all fibers receive simultaneous field stimulation over the course of the experiment. Frequency of stimulation was 10 Hz, in trains of 5 seconds every 50 seconds. Fibers not responding to electrical stimulation (failing to twitch) at the t + 90 time point were excluded. NFATc1 activation was estimated by the ratio of GFP signal in the nucleus to the GFP signal in the cytoplasm (N/C). The 60 minute period prior to stimulation was averaged together and used as a baseline to measure change after 90 minutes of stimulation (∆N/C). Due to heterogeneity in the baseline measurement, the ΔN/C was calculated for individual nuclei prior to averaging.

### Kinase inhibition

**Figure 3:**
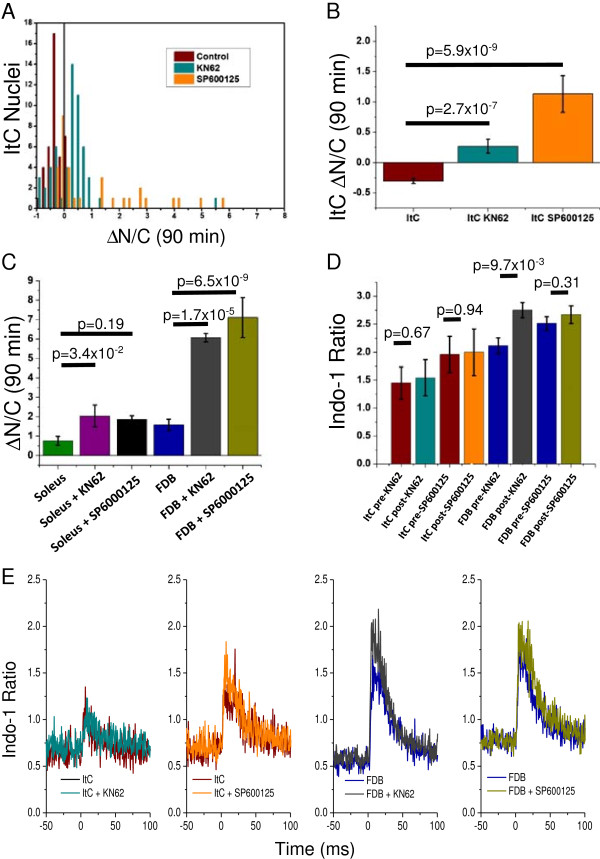
**Pharmacological inhibition of CaMKII and JNK.** Inhibition of either CaMKII and JNK is enough to restore activity dependent activation of NFATc1 in the intercostal fibers. **(A)** Number of nuclei vs N/C ratio after 90 minutes of stimulation (∆N/C(90 min)) relationship in intercostal fibers treated with KN62 (cyan), SP600125 (orange) and corresponding control (red). **(B)** Average ∆N/C(90 min) in intercostal fibers treated with KN62 (cyan), SP600125 (orange) and corresponding control (red). Note that this effect may be incomplete, resulting in activity-responsive subpopulations **(A)**, but nevertheless represents a significant reversal of the behavior observed in control intercostal fibers **(B)**. Similar treatment of FDB fibers results in significant increase in translocation during stimulation. **(C)** Average ∆N/C(90 min) in soleus and FDB fibers treated with KN62, SP600125 and corresponding control. Minor increases were also observed in soleus fibers but did not reach significance threshold. **(D)** Summary of peak indo-1 ratio measurements for intercostal and FDB fibers treated with KN62, SP600125 and corresponding controls. Treatment with kinase inhibitors does not reduce magnitude of indo-1 calcium transients. Averaged traces of indo-1 ratios are also shown **(E)**. Sample size **(A, B and C)** control n = 40, 37, 38 nuclei from 15, 14, 25 fibers isolated from 4, 4, 3 animals for ItC, Sol and FDB respectively; KN62 n = 56, 7, 4 nuclei from 18, 4, 2 fibers isolated from 5, 2, 1 animals for ItC, Sol and FDB respectively; SP600125 n = 32, 3, 9 nuclei from 11, 2, 4 fibers isolated from 4, 1, 1 animals for ItC, Sol and FDB respectively. Sample size **(D and E)** KN62 n = 3, 5 fibers for ItC and FDB respectively; SP600125 n = 3, 6 fibers for ItC and FDB respectively. Error bars show SEM.

In cases where pharmacological agents were applied, they were included in the media change to L-15 prior to imaging. The JNK inhibitor (SP600125) was applied at 500 nM (approximately five to ten times IC50 for JNK family members based on published estimations [25]). This concentration was selected to be significantly below the levels at which SP600125 inhibits most of the other kinases for which data is available to minimize off-target effects. The CaMKII inhibitor (KN-62) was applied at 5 μM based previous experiments in skeletal muscle fibers [22]. KN-62 was selected after our initial experiments with KN-93 eliminated the twitch response and calcium transients in isolated intercostal fibers (not shown). Due to concerns that KN-62 or SP600125 may impair the calcium transient, fibers were loaded with the ratiometric indicator indo-1 and calcium transients were measured. Control measurements were taken after 90 minutes of acclimation to the microscope. Inhibitors were then applied and measurements were repeated in the same fibers after 90 minutes of exposure (Figure 3).

### Western blots

Extracted muscles were ground with a pestle under TPER lysis buffer supplemented with protease inhibitors and kept on ice with periodic agitation for up to three hours until tendons were clean. Insoluble debris was removed by centrifuging samples at 4°C for ten minutes at 5,000 RPM. The supernatant was removed and concentration was estimated by Nanodrop-1000 spectrophotometer (Thermo Scientific, Wilmington, DE, USA. Approximately 30 μg per lane was denatured at 74°C for ten minutes and loaded onto precast 4 to 12% polyacrylamide gels under reducing conditions. After transfer, membranes were blocked in 5% milk with 0.1% Tween and then cut according to molecular weight so that samples could be normalized to proteins run in the same lane. Membrane sections were incubated overnight in primary antibodies. Primary antibodies were washed out then secondary fluorescent antibodies were applied for one hour and washed out. All antibodies used are commercially available as follows: α-actinin: 11 M4845, SigmaAldrich, St. Louis, MO, USA; JNK: SAB4200176, SigmaAldrich, St. Louis, MO, USA; CaMKII: sc-9035, Santa Cruz Biotechnology, Dallas, TX, USA; CN: 07–1491 Millipore, Billerica, MA, USA; secondary antibodies: a21235 and a21428, Life Technologies Invitrogen, Grand Island, NY, USA. Membranes were imaged on a Typhoon FLA 9500 biomolecular imager (GE Healthcare Life Sciences, Pittsburg, PA, USA). Bands were measured using ImageJ (NIH, Bethesda, MD, USA; http://rsb.info.nih.gov/ij/), following automated background subtraction. JNK/CaMKII/CN bands were normalized to the α-actinin band from the same lane and to the average of the ItC samples.

### Data analysis

After initial processing in ImageJ, data was handled in Origin 8 (OriginLab Corporation, Northampton, MA, USA). Significance of NFATc1 translocation was determined by Mann–Whitney *U*-tests due to significant deviations from normality in the data. Significance of measurements of JNK/CaMKII/CN was carried out by ANOVA, followed by pairwise unpaired *t*-tests. Significance of calcium measurements on kinase inhibited fibers was determined by paired *t*-tests. Bonferroni multiple comparison correction was used to adjust the significance threshold within each experiment. Data from Figures 2 and 3 were considered as a single set for the purposes of this correction due to the pooling of data from intercostal fibers with no pharmacological treatment.

### Animal use

All animals used in this study were housed and used in accordance with procedures approved by the University of Maryland Baltimore IACUC under protocol number 0412012.

## Results

### Reverse translocation

Our initial observation that NFATc1 showed abnormally high resting activation in cultured ItC fibers [24] leads us to examine the activity of NFATc1 in these fibers more closely. Fibers derived from both the soleus (Figure 2D-F) and FDB (Figure 2G-I) show increased nuclear import of NFATc1 characteristic of the canonical activation pathway following 90 minutes of repetitive electrical field stimulation. In contrast, the ItC fibers showed dramatic nuclear efflux of NFATc1 in response to the same activity pattern (Figure 2A-C). This effect was observed in the overwhelming majority of ItC nuclei (Figure 2K) and represents a significant difference from both FDB (*P* = 6.8 x 10^-20^, < 0.0125) and Sol (*P* = 2.7 x 10^-17^, < 0.0125) fibers undergoing similar treatment (Figure 2L).

### Inhibition of activity regulated kinases

A probable mechanism for the activity dependent inactivation of NFATc1 in the intercostal muscles is a comparatively high level of activity regulated kinases relative to the canonical pathway of CN, such that the canonical activity of CN is simply overwhelmed. If this is so, inhibition of these kinases should permit the canonical CN pathway to proceed, resulting in the more typical activity induced activation of NFATc1. Using the inhibitors KN62 and SP600125 (inhibitors of CaMKII and JNK, respectively), we find that antagonizing these kinases results in subpopulations (Figure 3A) which show significant activity dependent NFATc1 activation (Figure 3B) in response to both CaMKII (*P* = 2.7 x 10^-7^, < 0.0083) and JNK (*P* = 5.9 x 10^-9^, < 0.0083) inhibition. Both KN62 and SP600125 treated intercostal fibers maintain the ability to release calcium (Figure 3D) and twitch when subjected to electrical field stimulation (*P* > 0.0125). Similar treatment of Sol and FDB fibers had the anticipated result of increasing the level of translocation in response to stimulation, although only in the FDB fibers does the difference reach significance (*P* = 1.7 x 10^-5^, 6.5 x 10^-9^, < 0.0083 for KN62 and SP600125 treated FDB fibers respectively).

### Kinase/phosphatase expression levels

**Figure 4:**
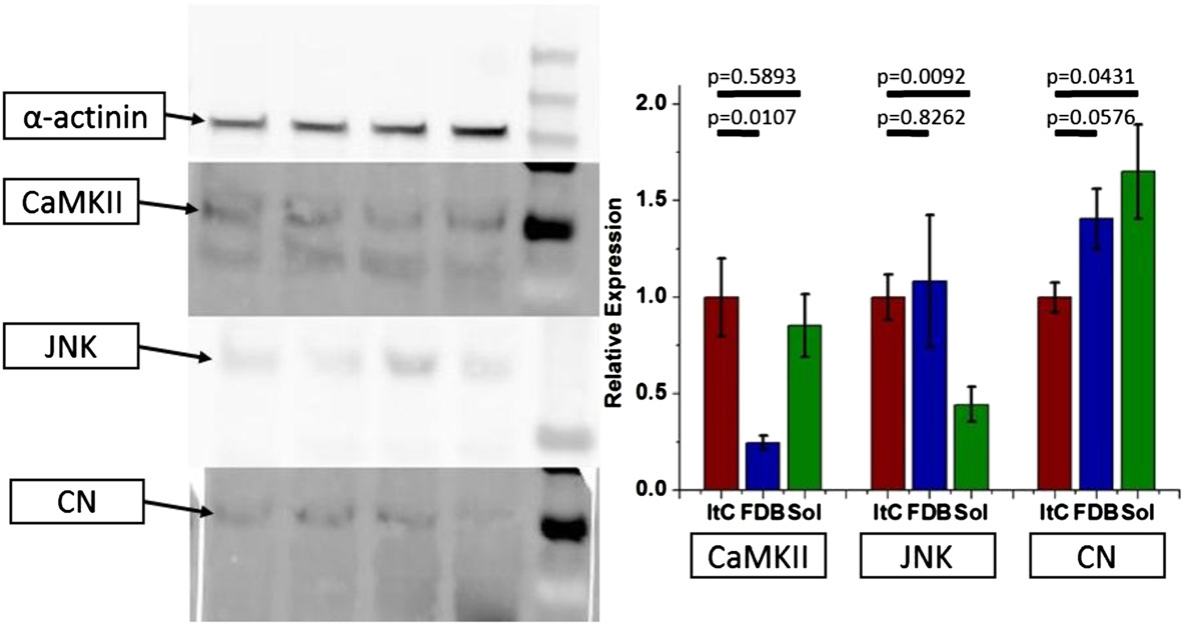
Western blots of CaMKII, JNK and CN.

One simple mechanism for controlling the activity of kinases and phosphatases in a sustained, muscle specific manner is to control the level of kinase/phosphatase present in each muscle. To examine this possibility, we estimated the relative expression levels of CaMKII, CN and JNK in lysates of FDB, Sol and ItC muscle. ANOVA showed significant dependence on muscle of origin in the expression levels of these proteins (Figure 4, *P* = 0.00909). *Post-hoc t*-tests showed changes just below the significance threshold between ItC and FDB in the level of CaMKII (*P* = 0.0107, > 0.0083) and between ItC and Sol in the level of JNK (*P* = 0.0092, > 0.0083) with no single protein appearing to account for the anomalous behavior of NFATc1 in ItC fibers at the level of raw expression.

## Discussion

### Overview

The role of NFATc1 in skeletal muscle plasticity and E-T coupling is critical [1]. While the core pathway has been thoroughly studied, this has previously been done in a limited variety of skeletal muscles which are routinely used to model skeletal muscles as a whole [24]. This has led us to overlook interesting phenomena in other muscles. The results reported here highlight the need to examine currently understudied muscles.

In this study we demonstrate a previously unreported activity dependent inactivation of NFATc1 in intercostal skeletal muscle fibers. This inactivation is CaMKII and JNK dependent, and may substantially alter the way in which the intercostal muscles adapt to physical activity compared with more commonly studied skeletal muscles.

### Reverse translocation

Our previously reported observation of elevated basal NFATc1 activation in isolated intercostal muscle fibers [24] spurred us to examine the activation of NFATc1 in this model. To our surprise, we found the canonical activation pattern reversed when we applied stimulation previously shown to strongly activate NFATc1 [8]. The nature of the isolated fiber model lead us to initially hypothesize that the basal levels of the activity-inducible kinases and phosphatases may be different in intercostal fibers compared with more commonly studied muscles. Changes in the relative levels of these proteins might de-emphasize the canonical role of CN and would persist in the absence of paracrine and nervous input for some time (factors which are largely absent in isolated muscle fiber cultures).

### Functional significance

Although our data are unable to support a full discussion of the functional significance of this phenomenon without examining the downstream transcripts under the control of NFAT, it does raise interesting implications. If NFATc1 behaves this way *in vivo* and no other pathways interfere, it would imply that endurance training of these muscles leads the muscle to transform toward the lower endurance fast phenotypes. Although the fiber types present in respiratory muscles are not frequently considered, they have been extensively studied in respiratory diseases such as chronic obstructive pulmonary disease (COPD). One tantalizing result from this work is that, as a result of continuous labored breathing, the intercostals in COPD have been observed to undergo a slow to fast transformation [26].

### Activity inducible kinase inhibition

The kinase activities of CaMKII and JNK are both increased by muscle activity. Work in other tissues has established that both kinases antagonize the activation of NFATc1 [12,23]. This makes them prime candidates to drive the unusual inactivation observed in the ItC muscle fibers. JNK has been shown in T-cells to phosphorylate NFATc1 directly on the CN targeting motif, preventing CN dependent dephosphorylation [12]. More recent work in cardiomyocytes indicates that CaMKII directly phosphorylates CN, inhibiting its activity [23]. Our results show that the activity dependent inactivation of NFATc1 in intercostal muscle fibers can be at least partially reversed by inhibition of either of these kinases, indicating that they both also play a significant role in this pathway in at least some skeletal muscles.

### Kinase/phosphatase expression levels

Our results do not clearly indicate one kinase/phosphatase as the key player behind the atypical behavior of NFATc1 in isolated intercostal muscle fibers. However we do show significant changes in the expression levels of relevant kinases/phosphatases between different types of muscle. While we do not rule out more transient mechanisms, we hypothesize that lineage dependent differences in expression levels of kinases and phosphatases in the NFATc1 activation pathway are the simplest and most plausible mechanism for the activity dependent NFATc1 inactivation reported here.

## Conclusions

The mechanisms coupling excitation to transcription in muscle are complex and delicate. Although we examine only a small part of the E-T coupling system here, we show clearly that the dominant mechanisms can and do differ from muscle to muscle. The completely opposite responses to identical activity patterns demonstrate a need for careful model selection in studying E-T coupling and highlight the role played by lineage in muscle type determination.

## Abbreviations

CaM: Calmodulin; CaMK: Calmodulin dependent kinases; CaMKII: Calcium/calmodulin dependent protein kinase II; CK1: Casein kinase 1; CN: Calcineurin; COPD: Chronic obstructive pulmonary disease; DYRK: Dual-specificity tyrosine-phosphorylation regulated kinase; E-T coupling: Excitation-transcription coupling; FDB: Flexor digitorum brevis; GSK3: Glycogen synthetase kinase 3; ItC: Intercostal; JNK: c-Jun NH2-terminal kinase; NFATc1: Nuclear factor of activated T-cells, calcineurin-dependent 1; Sol: Soleus; SP repeats: Serine-proline repeats; SRR: Serine rich region.

## Competing interests

The authors declare that they have no competing interests.

## Authors’ contributions

PR designed and carried out experiments, drafted and edited the manuscript and performed the statistical analysis. EOH contributed to experimental design, carried out several experiments on FDB fibers and edited the manuscript. MFS contributed to experimental design and edited the manuscript. All authors read and approved the final manuscript.
